# Response of Sugarcane in a Red Ultisol to Phosphorus Rates, Phosphorus Sources, and Filter Cake

**DOI:** 10.1155/2015/405970

**Published:** 2015-05-11

**Authors:** Gustavo Caione, Renato de Mello Prado, Cid Naudi Silva Campos, Leandro Rosatto Moda, Ricardo de Lima Vasconcelos, João Martins Pizauro Júnior

**Affiliations:** ^1^Mato Grosso State University, Alta Floresta, MT, Brazil; ^2^São Paulo State University, Jaboticabal, SP, Brazil

## Abstract

We evaluated the effect of phosphorus application rates from various sources and in the presence or absence of filter cake on soil phosphorus, plant phosphorus, changes in acid phosphatase activity, and sugarcane productivity grown in Eutrophic Red Ultisol. Three P sources were used (triple superphosphate, Araxa rock phosphate, and Bayovar rock phosphate) and four application rates (0, 90, 180, and 360 kg ha^−1^ of P_2_O_5_) in the presence or absence of filter cake (7.5 t ha^−1^, dry basis). The soil P, the accumulated plant P, the leaf acid phosphatase activity and straw, the stalk productivity, the concentration of soluble solids in the juice (Brix), the juice sucrose content (Pol), and the purity were the parameters evaluated. We found that P applications increased levels of soil, leaf, and juice phosphorus and led to higher phosphorus accumulation and greater stalk and straw productivity. These levels were highest in the presence of filter cake. Acid phosphatase activity decreased with increasing plant phosphorus concentration. Phosphate fertilization did not show effect on sugarcane technological quality. We concluded that P application, regardless of source, improved phosphorus nutrition and increased productivity in sugarcane and, when associated with filter cake, reduced the need for mineral fertilizer.

## 1. Introduction

Phosphorus (P) is essential for the synthesis of adenosine triphosphate and numerous other phosphorylated compounds [1]. This nutrient also enhances photosynthetic activity and increases root development, leading to increased nutrient uptake, greater tillering, and higher yield in sugarcane [2]. P deficiency in sugarcane induces biochemical change such that leaf phosphatase acid activity is negatively correlated with accumulated P [3]. This relationship could be used for early diagnosis of phosphorus nutrition in sugarcane.

Tropical soils have low available P due to low natural availability, clay adsorption, and precipitation with Fe and Al. Thus, the efficiency of phosphorus fertilization in cultivated soils is considered low. It is estimated that 85 to 90% of inorganic P added to the soil becomes unavailable to plants in the year of application [4]. The amount of P remaining in balanced solution after P application is called remaining P [5]. The concentration of remaining P depends on P application rate, contact time [6], and the phosphate adsorption capacity of the soil. This final factor depends on the amount of organic matter, clay texture, and clay mineralogy [7]. Thus, phosphorus fertilization should be managed to improve absorption by the plant, decrease soil adsorption, and consequently improve phosphorus usage by the crop.

Some studies indicate that the association of mineral phosphate fertilizer with organic compost leads to higher available soil P [4, 8–11], better plant absorption, and greater productivity [12]. Filter cake is an important organic compost and a by-product of sugar and ethanol plants that comes from the sugar clarification process and is composed of ground bagasse and decanted sludge. One ton of ground sugarcane produces 30 to 40 kg of filter cake [13]. As sugarcane production increases, filter cake production also increases, which can then be used to optimize crop fertilization. Filter cake can partially substitute mineral fertilizers [12, 14–16]. Specifically, applications of this organic compost at 15 t ha^−1^ (wet basis) can reduce conventional chemical fertilization by 50% [17]. Nevertheless, there is little information about the effect of this compound on sugarcane when associated with P sources and P application rates.

In general, studies on increasing sugarcane productivity as a function of phosphate fertilization have shown varying results [2, 13, 17–22]. However, variations in the magnitude of crop response could depend on the P sources and application rates and the presence of organic compost. Some studies show that filter cake increases soil pH [12], which could favor highly soluble P sources and limit lower solubility sources. Other authors did not show the same effect [23].

We evaluated the effect of P application rate, P source, and the presence or absence of filter cake on soil P, plant P, changes in leaf acid phosphatase activity, and productivity of sugarcane plants grown in Ultisol.

## 2. Material and Methods

### 2.1. Experimental Area

**Figure 1:**
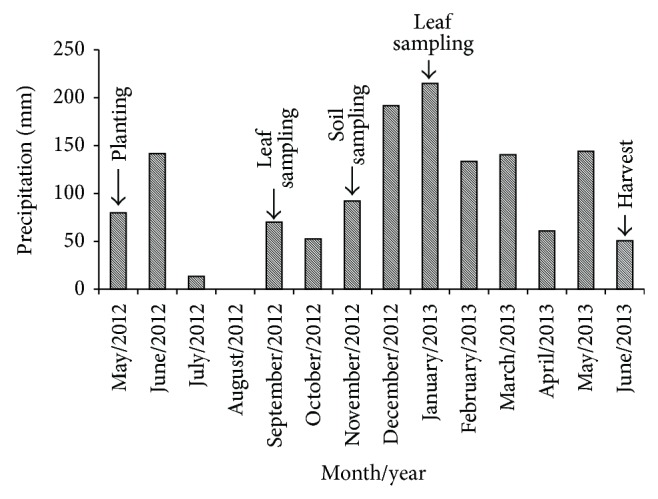
Monthly accumulated precipitation during the experiment. Source: [38].

The experiment was carried out in the field and during the 2012/2013 growing season and was located in Catanduva, São Paulo state, Brazil (21°05′07^″^S, 48°54′22^″^W, height 550 m). The predominant climate of the region is tropical rainy with dry winters (Aw, Köppen).  Figure 1 shows the precipitation data collected during the experiment. It can be seen that rainfall was well distributed (1385 mm total) and that there were no water limitations during the sugarcane growth cycle.

The soil in the experimental area was classified as Eutrophic Red Ultisol [24]. Twenty soil subsamples were collected from the 0–20 cm layer. Soil chemical analysis using the methodology of [25] showed the following: pH (CaCl_2_) = 5.5, organic matter = 12 g dm^−3^, P (resin) = 5 mg dm^−3^, K^+^ = 3.1 mmol_*c*_ dm^−3^, Ca^2+^ = 30 mmol_*c*_ dm^−3^, Mg^2+^ = 13 mmol_*c*_ dm^−3^, H^+^ + Al^3+^ = 18 mmol_*c*_ dm^−3^, sum of bases = 64 mmol_*c*_ dm^−3^, cation exchange capacity = 64 mmol_*c*_ dm^−3^, and base saturation of 72%. Fe_2_O_3_, Al_2_O_3_, and SiO_2_ levels (4.7%, 8.0%, and 11.2%) were measured according to the methodology described by [26].

### 2.2. Experiment Setup and Crop Treatments

The experimental area received lime applications five months prior to the start of the experiment. Thus, base saturation was adequate for sugarcane cultivation [27] and thus unnecessary to correct for soil acidity. The soil was prepared conventionally by plowing and harrowing in March 2012 and then leveling (by harrowing), furrowing (0.30 m depth), and planting in May 2012.

CTC 15 sugarcane was used. This variety is classified as medium/late cycle, highly productive, robust, drought tolerant, and adaptable to various production environments [28]. The sugarcane stalks were manually cut into 3-bud sets, planted end to end at an average row density of fifteen buds per meter, and then covered with a soil layer of approximately 0.10 m.

Fertilization at planting was carried out according to the recommendations of [27] except for variations in P and filter cake in accordance with the treatments. Treatments without filter cake were balanced with N (assuming 30% of the total N contained in the filter cake) and K (assuming 100% of the total K contained in the filter cake) at levels equal to those supplied by the filter cake. Ca and Mg levels were high throughout the experiment due to the liming.

Fertilizers were mixed and then applied at the base of the furrows. A nitrogen side dressing (50 kg ha^−1^ ammonium nitrate) was applied 40 days after planting [27]. Weeds were controlled with applications of tebuthiuron (1.2 L ha^−1^), ametryn (3.0 L ha^−1^), and MSMA (1.0 L ha^−1^). Pest and disease treatments were unnecessary.

### 2.3. Treatments and Experimental Design

The experiment was set up in randomized blocks of 3 × 4 × 2 with three repetitions representing 3 P sources triple superphosphate (41% soluble in 2% citric acid), Araxa rock phosphate (4% soluble in 2% citric acid), and Bayovar rock phosphate (14% soluble in 2% citric acid), four application rates of P_2_O_5_ (0, 90, 180, and 360 kg ha^−1^ of P_2_O_5_ soluble in 2% citric acid), in the presence or absence of organic compost (7.5 t ha^−1^ of filter cake, dry basis). The organic compost was obtained from decomposed filter cake and had the following chemical characteristics expressed in terms of dry matter at 60–65°C and using the methodology described by [29]: N = 14.0 g kg^−1^; P = 9.2 g kg^−1^; K = 3.4 g kg^−1^; Ca = 25.3 g kg^−1^; Mg = 9.0 g kg^−1^; S = 3.3 g kg^−1^; B = 16 mg kg^−1^; Cu = 43 mg kg^−1^; Fe = 9.374 mg kg^−1^; Mn = 753 mg kg^−1^; and Zn = 70 mg kg^−1^. Also, it was determined the C/N ratio and the pH value of the filter cake and the values were 12.1 and 8.2 respectively. Each plot was performed by five rows of sugarcane, with 15 m long by 1.5 m between rows. The useful area to collect data was composed by the 13 m of the three central rows.

### 2.4. Evaluations

Leaf samples (middle third of leaf +3, excluding midrib) were taken 4 months after sprouting and used to evaluate the nutritional state and P level of the crop [30]. At eight months, when the crop was fully developed, leaf samples were collected again (middle third of the leaf +1), excluding midrib [27]. Sample preparation and chemical analysis were conducted using the methodology described by [29].

Six months after commencement of the experiment, soil samples were taken at 12 random points in the middle three furrows (0–0.2 and 0.2–0.4 m deep) of each plot. P levels in the samples were determined by the resin method [25] and the remainder method [6].

Another leaf sample was collected 8 months after sprouting (middle third of the leaf +1, excluding midrib). These samples were stored in liquid nitrogen and then used to evaluate alternative nutrition, biochemistry, and acid phosphatase activity using an adapted version of the methodology described by [31]. After thawing, the leaves were homogenized in Turrax homogenizer (OMNI, model GLH -2511), in 100 mM acetate buffer, pH 5.5, and at a ratio of 1 g of plant tissue to 10 mL of buffer. The homogenate was centrifuged at 10,000 g for 10 minutes at 4°C. The supernatant was then aliquoted, frozen in liquid nitrogen, and stored at −70°C. The aliquots were then used to measure the enzymatic activity and protein concentration of the extract. Protein concentration was determined by fluorescence using a Qubit fluorometer (Invitrogen). Manufacturer specifications were followed and bovine albumin serum was used as a standard.

Twelve months after sprouting, stalk and straw (leaves and apical meristem) productivity, number of millable stalks, stalk diameter, and stalk length were measured. Stalk diameter (first internode above the stalk base) and length (after the cut) were determined from 10 stalks per plot. Stalk number was determined from a 2 m section in the center row of each plot. A three-meter section from each row was harvested and the stalks (t ha^−1^) and straw (dry weight) were weighed separately. Samples were taken from each fraction and dried in a forced air oven (63–67°C) until reaching a constant weight and dry mass. After drying, the samples were ground in a Willey mill and then P was measured in the stalks and straw. The results were then used to calculate accumulated P in stalks and straw. Juice samples were also collected and measured to determine P levels. The same methodology used to determine P levels in the leaf samples was also used for the stalks, straw, and juice.

During the sugarcane yield evaluation, ten contiguous stalks were sampled from the central lines of the plots in order to analyze sugarcane technological quality. The concentration of soluble solids in the juice (Brix), the juice sucrose content (Pol), and the purity were the parameters evaluated.

### 2.5. Statistical Analysis

The Sisvar application [32] was used to calculate analysis of variance and perform *F* tests. A Tukey test (*P* ≤ 0.05) was used to compare variable, P source, and filter cake averages. Polynomial regression analysis was used to evaluate P application rates. Microsoft Excel Starter 2010 in Windows 7 Starter was used to produce the graphs. Finally, simple linear correlation tests between variables were performed using Assistat software, version 7.6 beta [33].

## 3. Results

### 3.1. Soil and Plant Phosphorus from Phosphate Fertilization

**Figure 2:**
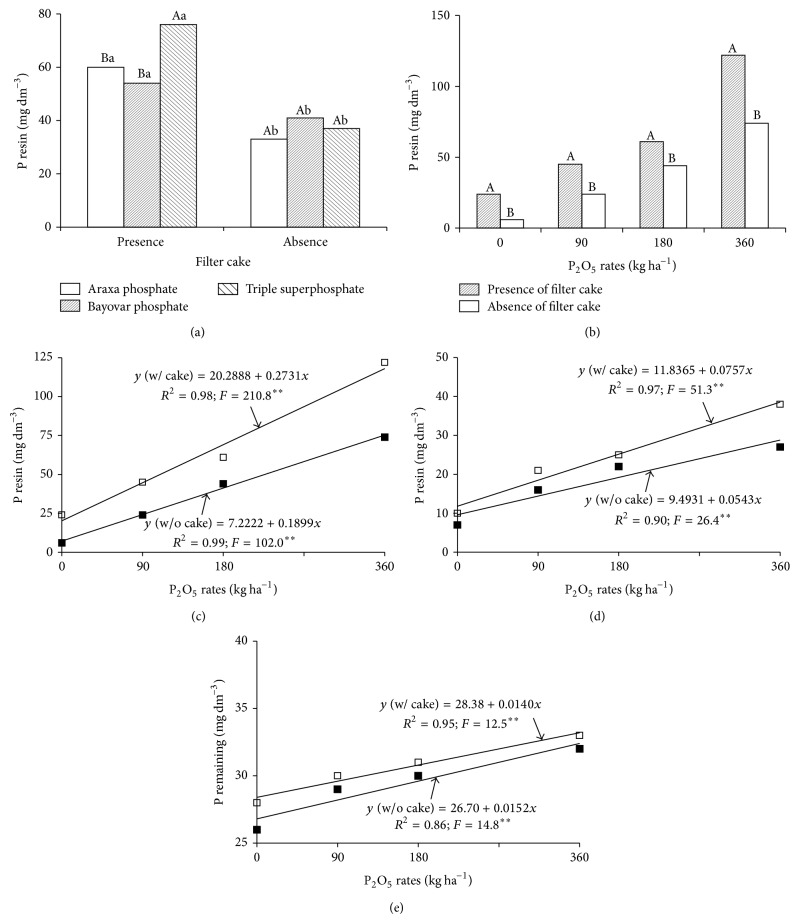
Available phosphorus (P resin) in the soil at 0.0–0.2 m, as a function of phosphorus source and filter cake (a), phosphorus application rate and filter cake (b and c) and depth 0.2–0.40 m, as a function of phosphorus application rate (d), and remaining phosphorus at 0.0–0.2 m, as a function of phosphorus application rate (e). Averages followed by single letters are significantly different (Tukey test, *P* ≤ 0.05). Capital letters compare P sources with the effect of filter cake. Lowercase letters compare the effect of filter cake on each source. ∗∗: significant (*P* = 0.01).

The P availability in the soil at a depth of 0.0–0.20 m, determined by the resin method, showed significant interactions between P source and filter cake and between application rates and filter cake (Table 1). In the presence of filter cake, triple superphosphate produced higher soil P than either Araxa or Bayovar rock phosphate; however, all sources showed high P content [27], regardless of the presence or absence of filter cake (Figure 2(a)). Higher levels from triple superphosphate may be caused by higher pH from the filter cake [12]. P levels were higher in the presence of filter cake than in the absence, regardless of source. All P application rates produced higher P resin in the presence of filter cake (Figure 2(b)). Application rate had a more significant effect in the presence of organic compost. Specifically, P level increases caused by application rate increases were 44% higher in the presence of cake than in its absence (Figure 2(c)).

P-res at 0.2–0.4 m was only affected by isolated factors (Table 1). Triple superphosphate produced higher available P than Bayovar rock phosphate. Nevertheless, all P sources produced available P levels within the accepted average range of 16 to 40 mg dm^−3^ [27]. Soil P was higher in the presence of filter cake than in its absence. Soil P increased linearly relative to application rate, regardless of the presence or absence of filter cake. However, in the presence of filter cake, the slope of the accumulated P line increased 39% for every unit of P applied relative to the increase in accumulated P caused by the application of P in the absence of filter cake (Figure 2(d)). This result is similar to that obtained at the 0.0–0.2 m depth.

Application rate affected remaining P only at the 0.0–0.2 m depth (Table 1) and rates caused linear increases regardless of the presence or absence of filter cake (Figure 2(e)).

**Figure 3:**
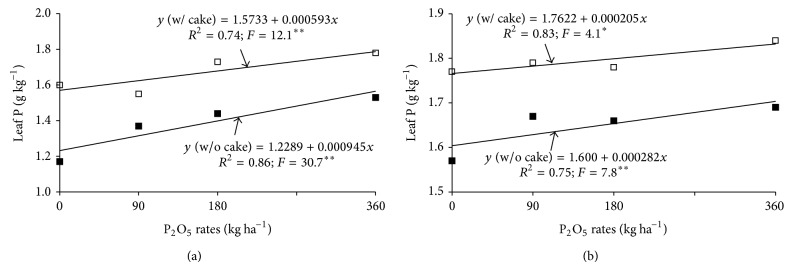
Leaf phosphorus in sugarcane at four months (a) and at 8 months (b), as a function of phosphorus application rate. ∗ and ∗∗: significant at probabilities of 0.05 and 0.01, respectively.

Leaf P at 4 and 8 months after sprouting was influenced by isolated factors but not by interactions (Table 1). Triple superphosphate produced higher P levels than Bayovar and Araxa rock phosphate at 4 months but no differences between sources were seen at 8 months. This result reflects the greater available soil P (P resin) at 0.2–0.4 m associated with this source. The presence of filter cake increased leaf P relative to its absence at both 4 and 8 months. This result is similar to the soil P result. Increasing application rates caused linear increases in leaf P, with or without filter cake. However, leaf P was always higher in the presence of filter cake at both 4 and 8 months (Figures 3(a) and 3(b)).

**Figure 4:**
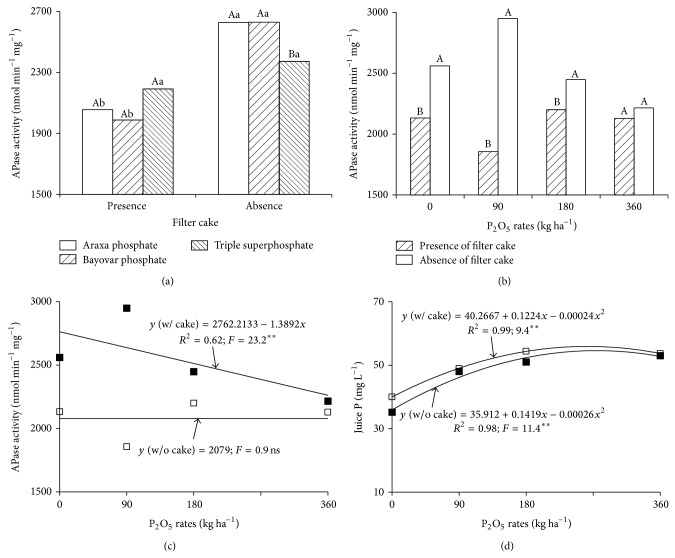
Acid phosphatase activity (APase), as a function of P sources and filter cake (a), P application rates and filter cake (b and c), and sugarcane juice P as a function of P application rates (d). Averages followed by single letters are significantly different (Tukey test, *P* ≤ 0.05). Capital letters compare P sources with the effect of filter cake. Lowercase letters compare the effect of filter cake on each source. ∗∗ and ns: significant (*P* = 0.01) and insignificant, respectively.

Interaction existed between P sources and filter cake and between application rates and filter cake regarding acid phosphatase activity (APase) (Table 1). Enzyme activity did not differ among P sources in the presence filter cake (Figure 4(a)). Nonetheless, triple superphosphate was associated with lower enzyme activity in the absence of filter cake. This result is related to higher available P in soil and leaves at four months, given that these enzymes are less active with higher cellular levels of inorganic P [34].

In the presence of filter cake, enzyme activity was lower with the use of Araxa and Bayovar phosphates but unchanged with triple superphosphate. Filter cake caused lower enzyme activity at P_2_O_5_ doses of 0, 90, and 180 kg ha^−1^ but had no effect on enzyme activity at 360 kg ha^−1^ (Figure 4(b)). This may be because phosphate nutrition was not improved by the presence of filter cake given the already high P level supplied at this application rate. In the absence of filter cake, P applications caused linear reductions in APase activity (Figure 4(c)) whereas the presence of filter cake had no effect on enzyme activity. This demonstrates that, without any P application (0 rate), the presence of filter cake had already reduced APase, which seems reasonable given that filter cake alone supplied adequate P as indicated by leaf samples (1.8 g kg^−1^ P) at eight months (Figure 3(b)), [27].

P applications increased P levels in the sugarcane juice. The highest juice levels resulted from the 255 kg ha^−1^ P_2_O_5_ rate in the presence of filter cake and the 273 kg ha^−1^ P_2_O_5_ in the absence of filter cake (Figure 4(d)).

### 3.2. Accumulated Shoot Phosphorus

**Figure 5:**
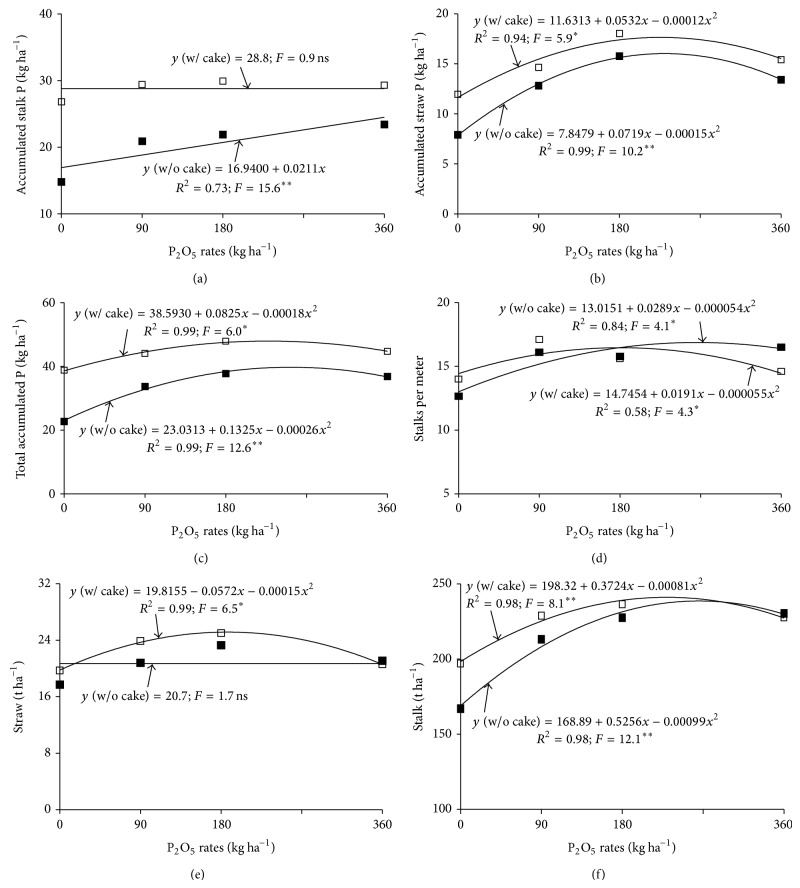
Phosphorus accumulation in stalks (a), straw (b), and total (c), number of stalks per meter (d), and productivity of sugarcane straw (e) and stalks (f) as a function of phosphorus application rate. ∗∗, ∗ and ns: significant at 0.01, 0.05 and insignificant, respectively.

Stalk, straw, and total accumulated P were influenced by application rate and by filter cake but not by source or interactions between factors (Table 2). The presence of filter cake produced higher accumulated P in stalks and straw and 39% higher total accumulated P than in the absence of filter cake. P applications in the absence of filter cake caused linear increases in accumulated stalk P (Figure 5(a)). At the highest rate, 24.5 kg ha^−1^ P was accumulated, which was just 7.6 kg greater than with no application (0 rate). In the presence of filter cake, P applications did not influence accumulated stalk P that was on average 28.8 kg ha^−1^ greater than the accumulation with the highest application rate and in the absence of filter cake. This result shows that, even at the 0-application rate, filter cake is an efficient P source for sugarcane.

### 3.3. Production, Productivity Components, and Sugarcane Technological Quality

The production components (stalk height and diameter) were not influenced by the treatments; however, stalk number was influenced by P application rate (Table 2). The greatest numbers of stalks (17 and 16 stalks per meter) were obtained at 174 and 268 kg ha^−1^ P_2_O_5_, in the presence and absence of filter cake (Figure 5(d)). Note that, when it was used filter cake the necessity of mineral P_2_O_5_ decreased by 94 kg ha^−1^ to obtain the maximum stalk number in comparison to absence of filter cake.

Only P application rate affected straw productivity (Table 2), which was 25.3 t ha^−1^ with 191 kg ha^−1^ P_2_O_5_ in the presence of filter cake (Figure 5(e)). Application rate had no effect on straw productivity in the absence of filter cake.

P application rate and filter cake were the only factors that had an isolated effect on stalk productivity (Table 2). Productivity was 6% greater with filter cake than without. This increase in productivity reflects the higher soil P and plant P and lower APase caused by the presence of organic compost relative to its absence.

P application rates, with or without filter cake, caused increases in stalk productivity that fit a quadratic model (Figure 5(f)). The highest productivity (241 t ha^−1^) with filter cake was obtained with a P_2_O_5_ application of 230 kg ha^−1^. Without filter cake, an extra 35 kg or 265 kg ha^−1^ P_2_O_5_ was needed to reach a maximum of 239 t ha^−1^. Therefore, the high correlation coefficient (*r* = 0.78^∗∗^) of stalk number showed that this variable had the greatest effect on productivity. Total accumulated P had the second greatest effect, underscoring the importance of phosphate fertilization on increased production. There was no effect of phosphate fertilization on the concentration of soluble solids in the juice (Brix) (mean = 16.9%), on the juice sucrose content (Pol) (mean = 14.3%), and also on the purity (mean = 84.6%).

## 4. Discussion

### 4.1. The Effect of Phosphate Fertilizers on Soil Phosphorus

Available P was highest when P applications were made with filter cake (Figures 2(c) and 2(d)). P availability in the soil is influenced by various factors such as soil texture, clay content, clay type, and soil organic matter. Thus, some authors attribute increased P availability in the soil to combined applications of P and organic compost [4, 8, 9, 11]. The quantity of P that remains in solution depends on the phosphate adsorption capacity of the soil, which in turn depends on the quantity of organic matter, clay texture and clay mineralogy [7], and P application rate. Lower organic matter levels mean greater surface exposure for P adsorption and consequent reductions in P-rem. On the other hand, lower levels of poorly crystalized Fe and Al oxides mean lower P adsorption and greater P-rem [5]. In the present study, increases in available P with filter cake application can be mainly attributed to the P supplied by this organic compost (containing 9.2 g kg^−1^ of P). The same effect was reported by [14] that observed soil P increases from 14 to 94 mg kg^−1^ after applications of filter cake exclusively (100 t ha^−1^). Increases in soil P resulting from the exclusive use of filter cake were observed in a vertisol [14] and also from a combination of filter cake with sugarcane bagasse in a Dystrophic Red-Yellow Oxisol (Typic Acrustox) [10]. An application of filter cake at 15 t ha^−1^ (wet basis) improved soil fertility [17] relative to conventional chemical fertilization, which is indicative of greater nutrient absorption by the plant and is reflected in greater productivity.

### 4.2. Effect of Phosphate Fertilization on Plant Phosphorus

Higher available soil P was associated with greater nutrient absorption by the plants, causing higher leaf P levels (Figures 3(a) and 3(b)), lower APase activity (Figures 4(b) and 4(c)), greater juice P levels (Figure 4(d)), and greater accumulation (Figures 5(a), 5(b), and 5(c)). Energy storage is consequently higher as P is essential for the synthesis of ATP and numerous other phosphorylated compounds [1]. This causes improved root development, tillering, and production [2]. The capacity for triple superphosphate fertilization to increase available soil P, plant absorption, and sugarcane productivity was also shown in Dystrophic Red-Yellow Oxisol (Typic Acrustox) [21] and Dystrophic Red-Yellow Ultisol [19]. Combining phosphate fertilizer with organic compost has been shown to result in increased phosphorus uptake by plants and higher productivity [12]. In the present study, P applications increased P levels in cane juice. The same result has been shown by other authors [35]. Higher P levels in sugarcane juice are important for commercial production because P helps clarify cane juice.

Increasing P applications caused linear increases in leaf P (Figures 3(a) and 3(b)) and linear reductions in APase activity (Figure 4(c)). Nevertheless, there was no significant correlation between APase activity and leaf P levels (*r* = 0.2 ns). Low levels of available P are reflected in plant enzymatic activity. Plants respond to P deficiency, with less efficient P usage and higher APase activity in leaves, stems, and roots [36]. Phosphatases are associated with P remobilization in plants. Therefore, increased activity of these enzymes has been linked to low cellular levels of inorganic P [34, 36] and a negative correlation between phosphatase activity and leaf P [37].

### 4.3. The Effect of Phosphate Fertilization on Productivity and Sugarcane Technological Quality

Phosphorus levels, especially in the presence of filter cake, increase levels of available soil P and plant uptake, which is reflected in higher productivity (Figures 5(e) and 5(f)); however, they did not show effect on sugarcane technological quality. Several studies have shown increases in sugarcane productivity as a function of phosphate fertilization [2, 13, 17–22]. Triple superphosphate applied to the furrow during planting (100 kg of P_2_O_5_) increased sugarcane productivity by 34% compared to a zero-application control [21]. Reference [22] evaluated the isolated effect of P applications in Nigerian soils with low P levels. Tsado et al. [22] found that applications of 150 kg ha^−1^ of P_2_O_5_ in the form of rock phosphate led to the highest stalk productivity 102.5 t ha^−1^ whereas a control treatment led to productivity of just 62.5 t ha^−1^.

Phosphorus benefits sugarcane in many ways. One way is by improving tillering, which has the greatest impact on sugarcane productivity [2, 18, 20]. Reference [18] showed that reducing mineral fertilization by 25% and adding filter cake at 15 t ha^−1^ can increase tillering by as much as 191% with consequent increases in stalk productivity. Authors state that the association between inorganic and organic fertilizers is very important for maintaining soil fertility and obtaining high sugarcane yields. Reference [14] only observed an increase in productivity from 73 to 85 t ha^−1^ with an application of filter cake (100 t ha^−1^). This result was confirmed by the present study in which the presence of filter cake increased soil and plant P (Table 1), which led to greater nutrient accumulation and greater stalk productivity (Table 2).

Thus, filter cake applications (10 t ha^−1^, wet basis) can reduce dependency on chemical fertilizers by as much as 25% [23]. Moreover, if the filter cake is enriched (15 t ha^−1^, wet basis) with* Azotobacter* and* Bacillus megaterium*, productivity can be increased by as much as 21% over chemical fertilization, potentially reducing chemical fertilizer requirements by 50% [17].

In general, many results show the effect of phosphate fertilization on yield increment; however, the technological quality is less affected. This fact can be explained by the influence of other yield factors, making the evaluation of fertilizers effects on those parameters difficult; therefore it might be related to genetic material [17, 18].

## Figures and Tables

**Table 1:** Soil phosphorus determined by the resin method (P-res) and by the remainder method (P-rem) at depths of 0.0–0.2 m and 0.2–0.4 m, leaf phosphorus at four months (P-4) and at eight months (P-8) after sprouting, and acid phosphatase activity (APase) and juice phosphorus (P-juice) of sugarcane grown in Eutrophic Red Ultisol, as a function of phosphate fertilization with source, application rate, and filter cake.

	P-res 0.0–0.2	P-res 0.2–0.4	P-rem 0.0–0.2	P-rem 0.2–0.4	P-4	P-8	APase	P-juice
	mg dm^−3^				g kg^−1^		nmol min^−1^ mg^−1^	mg L^−1^
P sources								
Araxa	47	20^ab^	30	19	1.4^b^	1.7	2340.9	47.8
Bayovar	45	18^b^	29	19	1.5^b^	1.7	2309.5	47.9
Triple superphosphate	57	25^a^	30	18	1.6^a^	1.7	2284.0	49.3
P_2_O_5_ rates								
0	15	8	27	19	1.4	1.6	2346.7	37.0
90	35	18	30	19	1.5	1.7	2402.7	48.9
180	53	24	30	18	1.6	1.7	2324.4	53.2
360	99	33	32	19	1.7	1.8	2172.6	53.7
Filter cake								
Presence	63	24	31	19	1.7	1.8	2079.5	49.5
Absence	37	18	29	19	1.4	1.6	2543.4	47.2

	*F* test							
Source	3.0^ns^	4.2^∗^	0.1^ns^	0.2^ns^	9.8^∗∗^	0.2^ns^	0.4^ns^	0.4^ns^
Rate	101.3^∗∗^	26.3^∗∗^	10.0^∗∗^	0.6^ns^	14.6^∗∗^	4.7^∗∗^	3.3^∗^	22.5^∗∗^
Filter cake	54.6^∗∗^	8.3^∗∗^	3.9^ns^	2.0^ns^	81.0^∗∗^	62.3^∗∗^	73.2^∗∗^	2.1^ns^
Source × rate	2.1^ns^	1.4^ns^	0.1^ns^	0.9^ns^	2.2^ns^	1.2^ns^	1.5^ns^	0.4^ns^
Source × cake	4.7^∗^	0.2^ns^	0.1^ns^	0.1^ns^	1.3^ns^	0.6^ns^	7.1^∗∗^	0.2^ns^
Rate × cake	4.5^∗∗^	1.0^ns^	0.2^ns^	0.4^ns^	2.7^ns^	0.9^ns^	16.6^∗∗^	0.4^ns^
Source × rate × cake	1.0^ns^	0.7^ns^	0.1^ns^	1.1^ns^	2.2^ns^	0.9^ns^	2.3^ns^	0.1^ns^

CV, %	29.9	40.4	10.6	6.5	8.9	4.7	10.0	13.7

Averages followed by single letters are significantly different from other averages in the same column (Tukey test, *P* ≤ 0.05). ∗∗, ∗ and ns: significant (*P* ≤ 0.01, *P* ≤ 0.05) and insignificant (*F* test).

**Table 2:** Stalk phosphorus accumulation (P-stalk) in leaves and apical meristem (P-straw), total (P-total), length (SL), diameter (SD), stalk number (SN), and the productivity of straw and stalks of sugarcane grown in Eutrophic Red Ultisol, as a function of phosphate fertilizer with different P sources, P application rates, and filter cake.

	P-stalks	P-straw	P-total	SL	SD	SN	Straw	Stalks
	kg ha^−1^ of P			m	mm		t ha^−1^	
P sources								
Araxa	24.8	13.1	37.9	3.4	28.5	15	21.2	217.4
Bayovar	23.5	12.8	36.3	3.4	28.7	15	21.3	209.7
Triple superphosphate	25.4	15.3	40.7	3.4	28.6	16	22.0	221.0
P_2_O_5_ rates								
0	20.8	9.9	30.7	3.4	28.4	13	18.7	182.0
90	25.2	13.7	38.9	3.4	28.6	17	22.3	221.0
180	25.9	16.9	42.8	3.5	28.5	16	24.2	232.0
360	26.3	14.4	40.7	3.3	28.8	16	20.8	229.1
Filter cake								
Presence	28.9	15.0	43.9	3.4	28.6	16	22.3	222.5
Absence	20.3	12.5	32.8	3.4	28.6	15	20.7	209.6

	*F* test							
Source	1.3^ns^	2.6^ns^	2.9^ns^	0.5^ns^	0.1^ns^	0.4^ns^	0.2^ns^	1.3^ns^
Rate	6.4^∗∗^	8.6^∗∗^	12.4^∗∗^	1.8^ns^	0.5^ns^	5.9^∗∗^	3.5^∗∗^	16.0^∗∗^
Filter cake	73.0^∗∗^	6.6^∗^	54.7^∗∗^	1.1^ns^	0.1^ns^	0.0^ns^	1.7^ns^	5.0^∗^
Source × rate	0.5^ns^	0.6^ns^	0.5^ns^	0.2^ns^	0.3^ns^	1.1^ns^	0.7^ns^	1.0^ns^
Source × cake	0.5^ns^	0.6^ns^	1.0^ns^	1.0^ns^	0.8^ns^	0.2^ns^	0.6^ns^	0.1^ns^
Rate × cake	1.6^ns^	0.3^ns^	1.4^ns^	0.7^ns^	1.3^ns^	2.2^ns^	0.4^ns^	1.4^ns^
Source × rate × cake	0.7^ns^	0.6^ns^	0.7^ns^	1.1^ns^	0.3^ns^	0.5^ns^	0.4^ns^	0.1^ns^

CV, %	17.4	29.3	16.7	5.3	3.5	14.9	24.1	11.4

∗∗, ∗ and ns: significant (*P* ≤ 0.01, *P* ≤ 0.05) and insignificant, respectively (*F* test).
